# Geoadditive models to assess spatial variation of HIV infections among women in Local communities of Durban, South Africa

**DOI:** 10.1186/1476-072X-10-28

**Published:** 2011-04-17

**Authors:** Handan Wand, Claire Whitaker, Gita Ramjee

**Affiliations:** 1 National Centre in HIV Epidemiology and Clinical Research, Sydney, Australia; 2 HIV Prevention Research Unit, Medical Research Council, Durban, South Africa

## Abstract

**Background:**

The severity of the HIV/AIDS epidemic in South Africa varies between and within provinces, with differences noted even at the suburban scale. We investigated the geographical variability of HIV infection in rural areas of the eThekwini Metropolitan Municipality in KwaZulu-Natal province, South Africa.

**Method:**

We used geoadditive models to assess nonlinear geographical variation in HIV prevalence while simultaneously controlling for important demographic and sexual risk factors. A total of 3,469 women who were screened for a Phase-III randomized trial were included in the current analysis.

**Results:**

We found significant spatial patterns that could not be explained by demographic and sexual risk behaviors. In particular, the epidemic was determined to be much worse 44 km south of Durban after controlling for all demographic and sexual risk behaviors.

**Conclusion:**

The study revealed significant geographic variability in HIV infection in the eThekwini Metropolitan Municipality in KwaZulu-Natal, South Africa.

## Introduction

South Africa is home to 5.7 million people living with HIV - the largest epidemic in the world [1], accounting for some 17% of the global HIV positive population. The reasons for the high rates of infection in South Africa remain uncertain, but socio-economic, cultural and historical factors are known to be important determinants of the spread of HIV infection [2-4]. HIV infection is not homogenously distributed throughout the South African population; population sub-groups are particularly vulnerable. The most vulnerable are young women between the ages of 25 and 29 years, 33% of whom are living with HIV [5], while the province of KwaZulu-Natal (on the eastern seaboard) has the highest HIV prevalence of the country's nine provinces at 26% (15-49 year age group) [5]. Other studies have demonstrated geographical variation in prevalence at the sub-provincial and even sub-district level [6,7].

Prevalence of HIV infection in South Africa has previously been reported as a national or provincial average [5]. Linking individual behavioral survey records with disease prevalence at community level has not previously been possible because of methodological challenges in existent methods. However, fine-scale geographical display and analysis of the data gathered during large-scale clinical trials could assist with public health policy and planning, allowing the allocation of scarce resources to areas most in need, and provide insight into the behavioral, demographic and socio-economic drivers of local epidemics.

The current study assessed geographical clustering in the region using flexible geoadditive models [8] in which the spatial component characterizes the correlation of the cases. This class of models allows us to measure small-area district-specific spatial effects simultaneously with possibly non-linear effects of other factors. The methodology assesses effects of spatial resolution which is not available with traditional parametric models, and results reveal evidence of how to reduce HIV prevalence by improving socio-economic and public health conditions. We hypothesized that HIV infection rates are not only influenced by socio-economic, demographic and sexual risk behavior variables but that they also vary considerably across regions and districts.

### Data and Methods

#### Study Sites

The analysis reported here is based on a group of 3,469 sexually active women who consented to screening for the "Methods for Improving Reproductive Health in Africa" (MIRA) clinical trial of the diaphragm for HIV prevention. Briefly, MIRA was an open-label, randomized controlled trial in HIV-negative, sexually active women conducted between 2003 and 2006 [9]. Clinical Research Sites located in two towns in eThekwini Metropolitan Municipality (EMM), namely, Umkomaas (in the south of the municipality) and Botha's Hill (to the west) were included.

Briefly, the main eligibility criteria included being sexually active, HIV negative at screening, willingness to provide written consent and follow study procedures, not pregnant with intention to maintain their non-pregnant status, and residing in and around the study area for a minimum of 1 year. The principle ineligibility was HIV infection, based on the results of two rapid tests at the screening visits (Determine HIV-1/2, Abbott Laboratories, Tokyo, Japan and Oraquick, OraSure Technologies, Bethlehem, PA, USA). At all visits, all participants received counseling on risk reduction and as many male condoms as desired. Counselors emphasized that condoms were the only known method to prevent HIV and STIs (at the time of the study), and condoms should be used for every act of sex. Women who were identified as HIV-positive at screening were referred to local health care facilities for care and support. Women who seroconverted during the trial remained in the study and were provided with ongoing counseling and referral to local health care facilities for further care at the end of the study. The protocol and informed consent forms of MIRA trial were approved by the Biomedical Research Ethics Committee of the University of KwaZulu-Natal. This study is registered with ClinicalTrals.gov, number NCT00121459.

#### Geographical Data

Details of participants' place of residence were collected on a locator information form at screening. Residential areas were captured onto a spreadsheet. Areas were grouped into clusters, and forwarded to the GIS Lab (Malaria Research Unit of the South African Medical Research Council, Durban) for mapping purposes. Verbal consent was obtained from all participants enrolled in our trials to collect GPS co-ordinates that related to their places of residence. Using locator information collected at screening, field staff visited the participant's place of residence. Once an appropriate satellite fix was acquired the co-ordinates were recorded on a hand-held GPS device, and a back-up hardcopy of the data was also created. Participant's confidentiality was ensured through the use of identifying numbers linked to GPS coordinate readings instead of names and addresses. At the end of each working day, field staff captured the coordinates digitally on a spreadsheet. This data was forwarded to the GIS Lab (Malaria Research Unit of the South African Medical Research Council, Durban) for mapping.

### Statistical Analysis

The raw data are longitude and latitude and the *logit *(i.e. logarithm of the odds ratio)-values from a fitted logistic regression model where HIV status of the women in the study was the primary endpoint. The raw data are difficult to visualize and interpret. We used geostatistical type models [10] to construct an image plot over the region of interest by mapping the mean of a response of interest, *y*, based on data (HIV_*i*_, latitude_*i*_, longitude_*i*_), 1≤ *i*≤ *n *where HIV_*i*, _measures occurrence of HIV cases in a particular location. This class of models extends generalized linear and additive regression in a semi-parametric fashion to simultaneously incorporate linear and nonlinear nonparametric effects of usual covariates, nonlinear interactions among them, and spatial effects into a geoadditive predictor. Such models are derived as the sum of semi-parametric components in the form of an additive model and a linear mixed effect model, respectively. The geographical information is given in spatially aggregated form, that is, we only know or use that a statistical unit lives in a certain geographical district or region.

The geoadditive models convert geographically referenced responses to maps by controlling for confounding effects of other covariates such as demographic, socio-economic, sexual risk behaviors and biological factors which are known to be associated with higher HIV cases regardless of other environmental exposures. Based on these considerations, we decided to apply models with a spatial component and several important socio-demographic, sexual and biological factors. We converted the geographically referenced responses to maps by controlling for confounding effects of the other covariates such as age, language spoken at home (English vs. Zulu/others), age at first sex, cohabitation with a sexual partner (yes/no), tested positive for herpes (HSV2) or gonorrhea (found to be associated with higher HIV cases at the screening visit regardless of other environmental exposures). Based on this methodology, image plots of the HIV cases were created as a bivariate function of longitude and latitude. It provides an informative summary of the geographical variation in prevalence of HIV infection over the region and, in particular, shows possible 'hot spots' of high HIV prevalence. Such hot spots, if found to be significant, are almost inevitably surrogates for unobserved or unknown covariates such as proximity to a source of exposure.

Even for perfect measures of infection cases, kriging alone will not properly address the question of environmental causality. For example, a region with higher sexually transmitted infections may also be more likely to have higher HIV infection rates. This study aims to investigate this problem by obtaining data on all other available attributes and accounting for them in the mapping.

Current study used the kriging routines based on the assumption that the parameter being interpolated can be treated as a regionalized variable. The extension of kriging, sometimes known as universal kriging (e.g. [11] and [12]), allows for the incorporation of covariates. The univariate and multivariate logistic regression analyses were undertaken, using the forward stepwise technique, to identify independent risk factors for HIV seropositivity. Candidate variables were entered into the model with a p-value less than 0.1. To demonstrate the importance of the geographical component we fitted the following models sequentially:

#### Model 1

Unadjusted

#### Model 2

Adjusted for age

#### Model 3

Adjusted for age + demographic risk/sexual risk category:

#### Model 4

Adjusted for age + demographic risk/sexual risk category + biological risk factors:

Where *f *is a real valued bivariate function, *β*_0 _denotes the intercept and β_*i*_, = 1,...,9 denote coefficients from logistic regression [i.e. log (odd ratio)] [13]. Based on this methodology, an image plot of the HIV cases were created as a bivariate function of longitude and latitude. Spatial components of the model *g (*latitude, longitude*)*_*j *_assumed to characterize correlations among the HIV cases. The area under the receiver operating characteristic (ROC) curve and the Hosmer-Lemeshow goodness-of-fit test were calculated to assess the performance of the final model.

Geoadditive models described in the previous section were fitted using the function semipar()in the statistical software system R.

## Results

The geographical data of a total of 3,469 women who were screened for the MIRA trial were used to investigate the spatial variation of HIV infections using geoadditive models. Top seven highest prevalences by district were presented in Table 1. The overall prevalence of HIV infection was 41%. Table 2 summarizes participants' characteristics by HIV seropositivity status. HIV prevalence was significantly higher among those who were aged 25-34 years old compared to younger (<25 years) or older (35+) groups (52% versus 38% and 30% respectively). HIV prevalence was 43% among women who reported speaking Zulu or other languages compared to 29% among those who reported speaking English at home (P < 0.001). Testing positive for HIV infection was more common among women who were not married (46%) and not living with a sexual partner (46%) compared to those married (14%) and living with a sexual partner (31%) (P < 0.001 both). High risk sexual behaviors were also common among those who were HIV infected at the screening visit compared with those not infected. More than 50% of the women who reported having had sex for the first time at age 14 or younger tested positive for HIV infection (P < 0.001). Sixty percent of women who had at least 4 or more lifetime sexual partners tested positive for HIV infection compared to those with less than 4 lifetime sexual partners (P < 0.001). Seventy percent of the women who reported having exchanged sex for money also tested positive. Prevalence of HIV infection was also significantly higher among women who tested positive for gonorrhea and Herpes (HSV2) (P < 0.001 both). HIV prevalence was significantly higher among women who reported not using any contraception methods (or using only traditional methods) compared to those who reported using at least one of the contraception methods (47% vs 40%, P < 0.001).

**Table 1:** Top seven highest HIV prevalences by districts

Districts in Study sites	Total tested for HIV	% infected
		
**Umkomaas (44 km South of Durban)**	**1,763**	**43%**

Emalangeni	12	83%
Amagcino	81	63%
aMahlongwa	159	56%
Amandawe	61	51%
Craigieburn	145	48%
Danganya	170	48%
Magabeni	176	43%
Others collectively	959	56%

**Botha's Hill (31 km West of Durban)**	**1,702**	**40%**
Mpumalanga	42	55%
Kwadabeka	45	49%
Clermont	56	46%
Inchanga	247	47%
KwaNdengezi	177	44%
Botha's Hill	51	43%
Hammarsdale	66	41%
Others collectively	1,018	64%

**Table 2:** Characteristics of the participants at the screening visit

Characteristics	**Total tested for HIV**^0^	**% infected**^†^	p-value
**Baseline age**			<0.001
<25 years	1,433	38%	
25-34 years	1,273	52%	
≥ 35 years	762	30%	
**Language spoken at home**			<0.001
English	413	29%	
Zulu/others	3,056	43%	
**Religion**			0.050
Christian	3,150	42%	
Others	318	36%	
**Marital status**			<0.001
Yes	523	14%	
No	2,946	46%	
**Cohabiting**			<0.001
Yes	1,053	31%	
No	2,416	46%	
**Age at first Sex**			<0.001
< 15 years	276	53%	
≥ 15 years	3,193	40%	
**Education**			0.633
Less than high school	3,422	41%	
At least high school	47	45%	
**Coital frequency (per week)**			0.702
≤3 times	2,709	41%	
>3 times	759	42%	
**Lifetime male sex partner**			<0.001
<4	2,551	35%	
≥ 4	918	60%	
**Tested positive for STI**^1^	555	46%	0.009
Tested positive for *T. vaginalis*			0.767
Yes	215	42%	
No	3,247	41%	
Tested positive for Gonorrhea			<0.001
Yes	99	64%	
No	3,363	41%	
Tested positive for *Chlamydia*			0.108
Yes	314	46%	
No	3,155	41%	
**Tested positive for HSV**			<0.001
Yes	2,523	52%	
No	946	13%	
**Exchange of sex for money**			<0.001
Yes	40	70%	
No	3,329	41%	
**Ever had sex using male condom**			<0.001
Yes	2,475	43%	
No	994	36%	
**Contraceptive use at screening**^2^			<0.001
Yes	2,701	40%	
No	767	47%	

^0^women with missing geographical data were excluded in the analyses, ^†^row percentages are presented

^1^At least one positive test for *Chlamydia*, *N. gonorrhea*, *C. trachomatis*, *T. vaginalis*, or *syphilis *at screening or enrollment

^2^Using any of the contraception listed below:

tubal ligation, vasectomy, intrauterine device, implants such as Jadelle and Norplant; combined oral contraceptive and progesterone only pills; male or female condoms

Table 3 presents the results from univariate and multivariate geostatistical models. Speaking Zulu (or languages other than English) at home, having first sex at age 14 or younger, not cohabiting with a sex partner, 4 or more lifetime sex partners, having a regular sex partner, testing positive for HSV2 or gonorrhea and contraception use were all significantly associated with HIV seropositivity at screening. In univariate analysis, estimated degrees of freedom for geographical component was 26.4 indicating a significant association between the HIV seropositivity and location at screening. Residuals from the final model fits were checked and showed no discernible patterns (data not shown).

**Table 3:** Univariate and multivariate analyses

Linear Component						
	**Univariate Analysis**			**Multivariate Analysis**		

**Covariate**	**Odds Ratio**	**95% CI**	**p-value**	**Odds Ratio**	**95% CI**	**p-value**

						
**Baseline age**						
≥ 29 years	1			1	1	
<29 years	1.18	1.03,1.35	0.019	1.15	1.01,1.33	0.041
**Language spoken at home**						
English	1			1		
Zulu/others	1.89	1.51,2.36	<0.001	1.50	1.18,1.91	0.001
**Marital status**						
Yes	1			-		
No	5.47	4.21,7.10	<0.001	-		
**Cohabiting**						
Yes	1			1		
No	1.91	1.63,2.22	<0.001	1.23	1.02,1.47	0.030
**Age at first Sex**						
< 15 years	1.67	1.30,2.13	<0.001	1.47	1.12,1.93	<0.001
≥ 15 years	1			1		
**Regular sex partner**						
No	1			1		
Yes	5.05	3.92,6.50	<0.001	4.69	3.52,6.26	<0.001
**Lifetime male sex partner**						
<4	1			1		
≥ 4	2.85	2.43,3.32	<0.001	2.35	2.00,2.78	<0.001
**Tested positive for Gonorrhea**						
Yes	2.55	1.68,3.86	<0.001	1.68	1.20,1.75	0.02
No	1			1		
**Tested positive for HSV**						
Yes	7.00	5.70,8.56	<0.001	6.65	5.36,8.24	<0.001
No	1			1		
**Exchange of sex for money**						
Yes	3.37	1.71,6.64	<0.001	-		
No	1					
**Ever had sex using male condom**						
Yes	1.38	1.18,1.60	<0.001	-		
No	1					
**Contraceptive use at screening**^1^						
Yes	1			1		
No	1.37	1.17,1.61	<0.001	1.45	1.20,1.75	<0.001
						
		Nonlinear component			Nonlinear component	
**Geographical component**	Degrees of freedom		knots	Degrees of freedom		knots
						
Longitude, Latitude	26.4		50	22.3		50

^1^Using any of the contraception listed below tubal ligation, vasectomy, intrauterine device, implants such as Jadelle and Norplant; combined oral contraceptive and progesterone only pills; male or female condoms

Although our primary concern in this study is geographical effects on HIV seropositivity, the non-linear covariate effect depicted here is quite interesting in its own right. Most importantly, the geographical estimates (Table 3) showed a significant association with HIV seropositivity. Above all, the nonlinear part of the geoadditive model was significant on the basis of the degrees of freedom.

**Figure 1:**
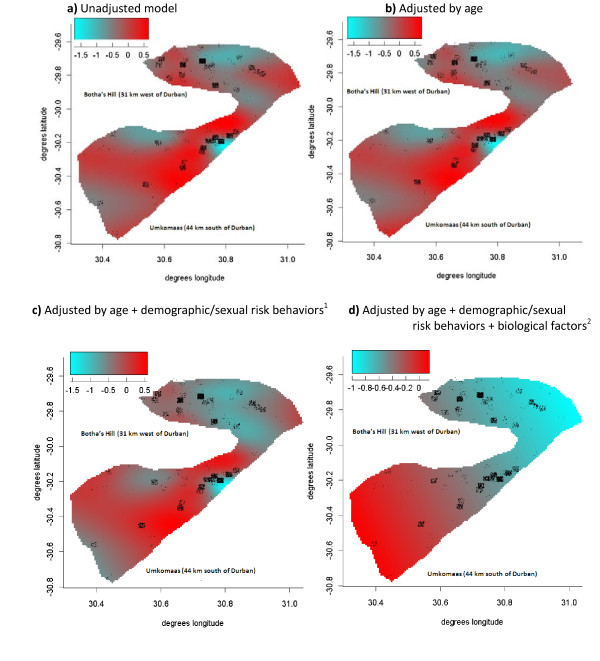
**Spatial variation of HIV infection in neighborhoods in the eThekwini Metropolitan Municipality**. ^1^language spoken at home, age at first sex, regular sex partner, 4 or more lifetime sexual partners, cohabitation status, contraception use, ^2^HSV, N gonorrhea

We also created crude (ignoring all the other risk factors) and adjusted maps over the region. In the analysis to create the crude map, the null hypothesis that HIV infection status does not depend on location (i.e. flat surface) resulted in estimated degrees of freedom of 26.4 for the geographical component, indicating a highly significant association between location and HIV infection status. Figure 1b shows the geographical variation of HIV infections adjusted for age (<29 vs ≥29). Estimated degrees of freedom of 25.2 for the geographical component still indicated a highly significant association between location and HIV infection status after adjusting for age. Figure 1c represents an attempt to control for age (<29 vs ≥29) and other demographic and sexual risk categories including language spoken at home (English vs. Zulu/other), cohabitation status, age at first sex <15, having a regular sex partner, having at least 4 or more lifetime sex partners, exchange of sex for money and contraception use. This analysis resulted in estimated degrees of freedom of 23.0 for the geographical component, indicating a highly significant association between location and HIV infection status even after adjusting for the risk factors which were known to be associated with HIV infection in the region. Finally, Figure 1d presents mapping of the final multivariate model (all the risk factors listed above as well as being diagnosed with HSV or gonorrhea at the screening visit) over the region. Results of the final multivariate model suggested that, considering the effects of the significant risk factors, the spatial distribution of HIV infections in the area is influenced above all by the geographical component. More specifically, Figure 1d shows a flat surface in the region around the Botha's Hill clinic (31 km west of Durban) compared to the region around the Umkomaas clinic (44 km south of Durban) demonstrating the presence of spatial confounding in the latter.

## Discussion

Investigating the spatial structure of HIV infection can have a profound impact on the epidemic dynamics, future spread, persistence and success of intervention. We provide here one of the first reports of spatial heterogeneity of HIV infection in the eThekwini Metropolitan Municipality (EMM) surrounding the city of Durban in South Africa, as measured using geoadditive models. We have identified an area of high prevalence in the south of the EMM which was not accounted for by variables measured in the study, and which may require further investigation to elucidate the causes and public health impact of the high prevalence of infection in this sub-region.

We have found that both marriage and cohabitation status influence the prevalence of HIV infection in this area of South Africa. The influence of marriage on HIV risk has previously been found to be somewhat variable [14-16]; however, in the South African context, it appears that marriage may be a protective factor [17]. In a similar trend, we found that cohabitation with a sexual partner offered some protection, and this effect was sustained in multivariate analysis. Studies of the influence of migration on HIV acquisition have indicated that disruption of a partnership poses a significant risk [18,19] - separation of partners, due to migration or because they cannot live together, may be associated with extra-pair concurrent partnerships.

The significance of several other previously recognized risk factors was reiterated in the multivariate analysis: testing positive for an STI (*Neisseria gonorrhoea *or HSV2) was significantly associated with HIV infection [20-22], as was having multiple lifetime sexual partners [17,23], and young age at sexual debut [24,25].

Geostatistical analysis is still somewhat underutilized as a tool for the identification and study of areas of high HIV prevalence in South Africa. Geostatistical analyses have previously been conducted for areas in northern KwaZulu-Natal [6,26], central and eastern districts of the Limpopo province [27,28], and the country as a whole [7]. Marked clustering or gradients of HIV prevalence (or mortality as proxy) were noted in all cases. In the case of northern KwaZulu-Natal, high HIV prevalence was associated with high density settlements near a national (primary) road; low HIV prevalence was associated with living in a more remote, less densely settled area [6]. High prevalence communities were wealthier and better educated than low prevalence communities, but had lower levels of marriage [6]. In our case, national roads run through both communities, with a more heavily trafficked route running inland near Botha's Hill to the west of the EMM which ultimately connects with the city of Johannesburg. The differential HIV prevalence in the south of the EMM is thus not explained by the presence or absence of a primary traffic route in the two areas under investigation. In an eastern district of Limpopo province, clustering of high mortality was associated with the presence of former Mozambican refugees, who were relatively more impoverished than residents of South African origin [27]. In the case of the central Limpopo district, the authors were restricted to speculating that high mortality was associated with close proximity to more urban areas, and greater distance from a health facility [28]. A country-wide mapping investigation showed that HIV prevalence was highest in eastern portions of the country (primarily KwaZulu-Natal), and declined towards the west, but high degrees of variability were also found within provinces [7]. Collectively, these studies indicate that a wide range of intersecting factors may impact upon the spatial distribution of HIV infection in South Africa, and that analysis of locally-derived data is of critical importance in developing district-specific strategies for tackling the epidemic. Although our analyses were based on HIV prevalence data, this should not preclude the cautious extrapolation of our results to HIV incidence for planning purposes; Tanser *et al*. expected that HIV incidence should follow HIV prevalence quite closely, since an individual's risk of acquiring the infection will be closely related to the level of infection in the surrounding population [6].

Geoadditive models combine the ideas of geostatistical and geoadditive models [10]. However, several other studies have treated the same structure in different ways for generalized responses [29-31]. Particularly, the extension of geoadditive models to survival data using both geographical point data and count data has received considerable attention since 2003 [32,33]. Geoadditive models with missing data are also studied by developing models that allow for specification of the covariate distribution and the missing data mechanism [34]. Current study has adapted and extended the methods of logistic regression models by incorporating the non-linear effect of a geographical component. Geoadditive regression models, where the spatial component is specified through stationary Gaussian random fields common in geostatistical methods offer a flexible approach for simultaneously exploring the impact of linear and non-linear covariate effects as well as of geographical effects, when the location of individual or statistical units can be observed. The general area of application is the spatial mapping of disease prevalence in settings where registry data are unavailable within a relatively small number of scattered communities.

Our study has some limitations that need to be considered in the interpretation of our results. First, because of the nature of the research conducted in these trials, populations selected were known to be at moderate-to-high risk of HIV infection because HIV incidence was expected to be sufficiently high to ascertain the efficacy of the intervention. The MIRA trial was able to target women from primarily rural communities of the EMM, therefore the women in this study may not necessarily be representative of women in KwaZulu-Natal province as a whole. Second, we cannot completely rule out the possibility that our findings may be due in part to unmeasured characteristics such as multiple or concurrent sex partners and commercial sex work. No data concerning migration of women or their partners were collected or included in these analyses. We were also unable to collect any sexual behavior data from male partners of the women. Nevertheless, the models were adjusted for high risk sexual behaviors such as increased frequency of sex. Third, STIs and pregnancy are evidence of unprotected sex; it is not clear whether these act as true risk factors for HIV acquisition or whether they are a proxy for having more unprotected sex. Nonetheless, baseline STIs were also associated with increased risk of HIV infection.

The results indicate that most HIV infections were accounted for by variables measured in the study. However, infections occurring in the south of the EMM could not be explained by measured variables, and must be associated with an unknown factor/s. It is possible, although speculative, that an economic influence may be at play - Botha's Hill lies between two cities, Pietermaritzburg and Durban, and is close to a major traffic route, while Umkomaas, although also lying on a national road, is not situated between major urban centres, and traffic density is reduced. Umkomaas is also closer to the boundary of the EMM, and participants attending the clinic may have travelled from areas outside the municipality which were less well resourced in terms of health care facilities and economic opportunities. Further research in this geographic area will be necessary to fully explain the observed excess of HIV infections.

## Competing interests

The authors declare that they have no competing interests.

## Authors' contributions

GR was a  co-investigator on the MIRA study. HW performed the statistical analysis. HW, CW and GR interpreted and drafted the manuscript. All authors read and approved the final manuscript.
